# Impacts of Air Velocity Treatments Under Summer Conditions: Part III—Litter Characteristics, Ammonia Emissions, and Leg Health of Heavy Broilers

**DOI:** 10.3390/ani14233525

**Published:** 2024-12-06

**Authors:** Derek West, Suraiya Akter, Bin Cheng, Edgar Oviedo, John Classen, Lingjuan Wang-Li

**Affiliations:** 1 Department of Biological and Agricultural Engineering, North Carolina State University, Raleigh, NC 27695, USA; drwest@ncsu.edu (D.W.); sakter@ncsu.edu (S.A.); chengbin0228@gmail.com (B.C.); classen@ncsu.edu (J.C.); 2 Prestage Poultry Science Department, North Carolina State University, Raleigh, NC 27695, USA; eooviedo@ncsu.edu

**Keywords:** broiler production, air velocity, NH_3_ emission, litter characteristics, leg health, FPD, HB

## Abstract

The broiler industry is crucial for rural economies, but it faces challenges in enhancing animal performance and well-being, particularly due to heat stress and ammonia (NH_3_) in grow-out houses. This study investigates the effectiveness of air velocity (AV) treatment in reducing heat stress and NH_3_ emissions while also improving litter conditions to enhance animal welfare. Conducted at the North Carolina State University’s poultry engineering laboratory, the research used six identical controlled chambers, exposing broilers to High and Low AV treatments from 28 to 61 days across four flocks during the summers of 2017 and 2018. The nitrogen mass balance (NMB) method quantified NH_3_ emissions, while litter quality was assessed for moisture content, pH, total nitrogen, and total ammoniacal nitrogen. Additionally, leg health was evaluated through assessments of footpad dermatitis (FPD) and hock burns (HB) at 42 and 61 days. Results showed higher NH_3_ emissions in Low AV chambers, while High AV conditions improved litter quality and resulted in greater nitrogen retention. Broilers in High AV chambers also had higher individual body weights and better leg health scores. Thus, optimizing AV effectively mitigates heat stress, controls NH_3_ emissions, and enhances overall broiler performance and welfare, offering insights for sustainable industry practices.

## Simple Summary

As climate change increases the incidence and severity of heat waves in the broiler-producing regions, the need to improve the effectiveness of heat removal systems in tunnel-ventilated houses will increase. This paper reports a part of a comprehensive investigation of air velocity (AV) effect on broiler performance and well-being as a way to improve the engineering design of the “air deflector” technique for cooling large broilers within existing broiler houses. This controlled live broiler heat stress chamber study examined the impacts of AV treatments on litter characteristics, ammonia emission, broiler leg health, and individual weight gain. The results revealed the significant improvement of litter quality, ammonia emission, and broiler leg health through enhancing AV at the bird level condition. Moreover, the study provides needed knowledge about dynamic AV treatment settings that will lead to enhanced broiler performance and welfare in response to different air temperatures and broiler ages.

## 1. Introduction

Heat stress exerts a substantial impact on the welfare parameters of commercially reared heavy broilers, encompassing both physiological and environmental dimensions [1,2,3,4,5,6,7]. Physiologically, elevated temperatures induce stress responses in broilers, adversely affecting leg health [1]. The intensified heat prompts reduced physical activity, leading to compromised muscle development [8,9] and bone strength in their legs [10]. Parameters such as hock burns (HB) and footpad dermatitis (FPD) are crucial indicators of leg health, reflecting the prolonged contact of hocks or footpads with wet litter [11,12,13]. The inverse correlation between HB and FPD underscores the need for comprehensive litter management strategies to enhance overall broiler welfare and performance. Furthermore, the implications of severe FPD extend beyond leg health, impacting broiler mobility, feed and water consumption [14,15], and the ability to dissipate heat. As broilers grow larger, their tendency to remain seated regardless of leg health [16,17] scores highlights the critical role of proper litter management in fostering optimal broiler performance and welfare.

Additionally, the environmental impact of heat stress manifests in the characteristics of the litter—the bedding material on the poultry house floor. Heightened temperatures elevate the birds’ water intake and excretion, resulting in increased moisture levels in the litter. The ensuing damp conditions foster FPD [18,19]. As the cost of bedding material continues to rise, and its availability remains limited, the common practice of reusing built-up litter over multiple flocks has become prevalent [20,21,22]. However, this practice, while economically driven, poses a potential threat to broiler welfare and overall growth due to failure of managing litter quality over time [23]. Because reused litter is often still wet from the previous flock and retains nitrogen (N) from excreta, which constantly releases NH_3_ and may adversely affect incoming broilers at the start of the next flock if not properly managed [24,25]. The consequences of improperly managed NH_3_ levels at the onset of a new flock can significantly reduce weight gain and overall welfare [26]. In addition to NH_3_ concerns, the relationship between ventilation rates, litter moisture content (M.C.), pH, and total nitrogen (TN) further complicates the understanding of proper litter management.

Environmental parameters such as air temperature (T), relative humidity (RH), and air velocity (AV) significantly impact the production, health, and welfare of broiler chickens [27,28]. As the industry increasingly seeks sustainable and effective solutions, there is a pressing need for focused research to enhance airflow at the bird’s level. Such enhancements can help mitigate heat stress and develop strategies to reduce in-house NH3 levels, particularly for heavy broilers. Increasing AV has been shown to be an effective approach to improving broiler performance and welfare [29]. Higher AV reduces litter M.C., which minimizes the impact of wet litter on the plantar surface of the footpad and hock. Moreover, higher AV decreases the effective T perceived by broilers in the house, allowing them to allocate energy toward feed and water consumption rather than dissipating heat.

Although the relationship between broiler environment conditions and litter characteristics has been studied, further research is needed to understand the specific impacts of AV treatment on litter characteristics, NH_3_ emissions, and heavy broiler leg health. Therefore, this study aims to evaluate the effectiveness of increased AV in enhancing broiler performance by reducing litter M.C. and improving overall litter quality.

The research was designed to address two key questions:

1. Can increasing AV in mechanically ventilated broiler production houses improve litter parameters (e.g., M.C., pH, total nitrogen) to reduce NH_3_ emissions?
2. Can increased AV promote better broiler performance and leg health?

By addressing these questions, the study contributes valuable insights to inform practical and sustainable approaches for optimizing broiler production systems.

## 2. Materials and Methods

All live bird experimental procedures relating to birds in the present study were approved by the North Carolina State University (NCSU) Institutional Animal Care and Use Committee (IACUC Protocol#: 16-279-A).

### 2.1. Bird’s House and Management

**Figure 1:**
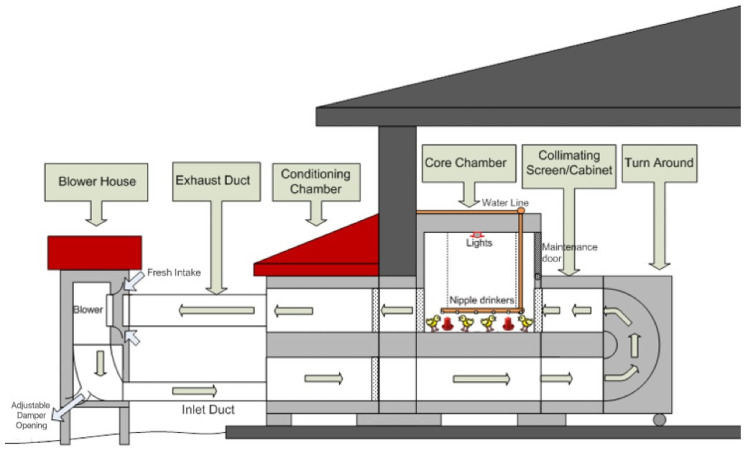
Side profile of a chamber system (from Shivkumar [32], used with permission).

Six identical chamber systems with the core chambers in dimensions of 2.44 m × 2.44 m × 2.44 m at the Poultry Engineering Chamber Complex at NCSU were used in this research to mimic commercial broiler grow-out conditions. As shown in Figure 1, each of the chamber systems was equipped with a variable frequency drive (VFD) to provide various ventilation rates and achieve various AVs at bird height in the range of 0.5–4 m/s according to bird age and air conditions. Each of the core chambers was equipped with a waterline with six nipple drinkers, 4 feeders, and 2 soft-white 40-watt light bulbs with an automatic switch-on timer. Each chamber’s lights were controlled electronically by dial timer switches, which were set to be on between 05:00 and 23:00 (5:00 AM and 11:00 PM). More details are available by [16,30,31].

Three different formulas (e.g., grower, finisher, and withdrawal) of pelletized feed were supplied to birds during each live bird experiment. The drinking water supplied to birds during grow-out in the chambers was sourced from a nearby groundwater well and distributed to birds via PVC piping and 6-nipple drinker lines per chamber. Two to three gallons of water were bled from each chamber’s drinking system prior to the start of each experiment, ensuring the water supply was fresh and free of debris. Drinker lines were checked daily to maintain proper functionality. Moreover, drinker line heights were raised accordingly in response to increasing broiler height.

Fresh pine wood shavings were placed in chambers prior to the start of each experiment. Three bags (for a total weight ranging from 22.89 kg to 24.45 kg, or 0.358 kg/m^2^) were used in each chamber for experiment 1, and only two bags (for a total weight ranging from 17.08 kg to 19.22 kg) were placed in each chamber for experiments 2–4.

### 2.2. Animals

Before being placed in chambers, 400 male broilers (ROSS 708) were hatched and raised in floor pens at the NCSU poultry unit under similar conditions. Among them, 264 birds without leg defects were randomly selected and placed in the chambers with 44 birds per chamber at 28 days of age. After a 7-day acclimation period at the age of 35 days, AV treatments commenced, and the birds remained in the chambers until they reached 61 days, adhering to a final stocking density of ≤40 kg/m^2^ to meet animal welfare regulations.

Four experiments were carried out for four flocks of birds in the summers of 2017 and 2018, and the details are given in Table 1.

During each experiment, chambers were checked for mortalities each morning at 7:00 am and periodically thereafter until each evening at 9:00 pm. The position and gesture of mortalities were photographed upon discovery, and the mortalities were weighed prior to being placed in plastic bags, frozen, and recorded in a mortality data log sheet.

### 2.3. The Core Chamber Environmental Monitoring

In each core chamber, environmental conditions (i.e., T and RH) were continuously monitored by different sensors described in previous literature [16,30,31]. Calibrated thermocouples placed in the inlet and outlet of each chamber measured and collected T at 1 min intervals while the HOBO Pro v2 External T/RH Data Logger, Model U23-002 (Onset, Computer Corporation, Bourne, MA, USA), stored T and RH at 10 min intervals. Details of the calibration and measurement are available in previous literature [11,19,20].

### 2.4. Air Velocity Treatments

Two sets of dynamic AV treatments were applied on birds during experimentation: High AV and Low AV. Treatments were characterized by bird age and the chamber inlet air T. Table 2 lists the two AV treatments; the difference varied in range depending on the bird’s age and how far the measured chamber T differed from optimal thermal conditions. During each of the experiments, AV treatments started on day 35 after birds moved into the chambers for a week. Details of the AV are given in previous literature [16,30,31].

### 2.5. Litter Sampling and Characteristics Analysis

**Figure 2:**
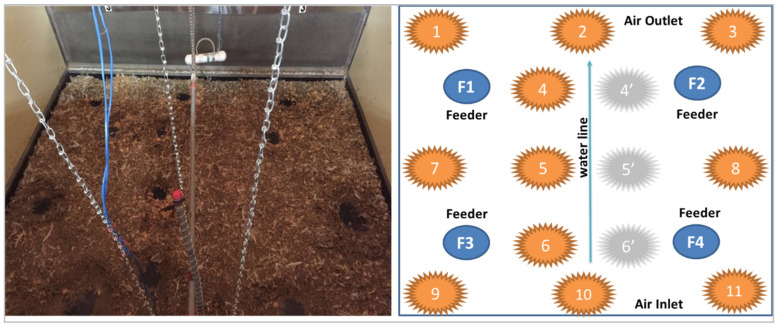
Illustration of spatial litter sampling locations in one of the core chambers (bird view) (The numbered circles are the location of litter sample collection).

Litter samples were collected from 15 locations (Figure 2) in each core chamber at the end of each experiment to capture spatial variation of TN, total ammoniacal nitrogen (TAN), M.C., and pH in litter. It was assumed that litter characteristics at locations across the drinker line are similar; therefore, litter samples were only taken on one side of the drinker line. The collected samples were then analyzed for the targeted characteristics at the NCSU Environmental Analysis Laboratory (EAL).

Area-weighted average (AWA) for all four characteristics (TN, TAN, M.C., and pH) of litter were assessed using the following formula to compare between two AV treatments and among four experiments:

(1) $$AWA=\sum_{i=1}^{15}\frac{A_{i}\times C_{i}}{A}$$

where AWA = area-weighted average of litter characteristics

A_i_ = area litter sample

C_i_ = measured TN, TAN, M.C., and pH litter sample

A = floor surface area of the core chamber (i.e., 8 ft by 8 ft, 64 ft^2^).

Unpaired, unequal *t*-tests were performed on AWA between AV treatments. Additionally, ANOVA tests, generated in R, were implemented to check for significant differences in AWA litter characteristics among experiments within the same AV treatment.

### 2.6. NH_3_ Emission Determination

Ammonia emissions were derived for each core chamber using the following nitrogen mass balance (NMB) Formulas (2) and (3).

(2) $$N_{lost}=(N_{\mathrm{bedding}\;\mathrm{material}}\times M_{\mathrm{bedding}\;\mathrm{material}}+N_{chicken-in}\times M_{chicken-in}\;+\;N_{feed}\times M_{feed})-(N_{mortality}\times M_{mortality}+N_{chicken-out}\times M_{chicken-out}\;+\;N_{litter}\times M_{litter})$$

(3) $${ER}_{{NH}_{3}}=N_{lost}\times [\frac{17}{14}]$$

where N_lost_ = mass of N lost to air through NH_3_ emission;

N_bedding material_ = N content of bedding material placed in the chamber in the beginning

M_bedding material_ = mass of bedding material placed in the chamber in the beginning of the experiment;

N_chicken-in_ = N content of chickens entering the chamber;

M_chicken-in_ = mass of chickens entering the chamber;

N_feed_ = N content of feed intake;

M_feed_ = mass of feed intake;

N_mortality_ = N content of mortalities;

M_mortality_ = mass of mortalities;

N_chicken-out =_ N content of chickens leaving the chamber at the end of the experiment;

M_chicken-out_ = mass of chickens leaving the chamber at the end of the experiment;

N_litter_ = TN content of litter (bedding plus manure) leaving the chamber at the end of the experiment;

M_litter_ = mass of litter (bedding plus manure) leaving the chamber at the end of the experiment;

ER_NH3_ = NH_3_ emissions in mass per day;

d = numbers of days of each experiment;

17/14 = the ratio of molecular weights of NH_3_ to N.

Fresh bedding material (wood shavings) was collected prior to the start of each experiment by ensuring a representative sample for the entire floor surface of each chamber. The same was litter samples were collected at the end of each experiment. Bedding material samples and litter samples were analyzed at the EAL for N and M.C.

In each experiment, five chicks among the 400 hatched were selected and euthanatized on the first day of each experiment for N analysis. These five euthanized broilers were frozen and ground by an industry-graded grinder at the Animal and Poultry Waste Management Center (APWMC) of NCSU. Grinded “meat” samples were analyzed for N content measurement at EAL. At the end of each experiment, three birds from each chamber were collected for N content analysis at EAL. The mortalities were also analyzed by EAL for the same parameters.

The weights of feed consumption of each formula were recorded, and feed samples were collected for each of the three types of feed. Feed samples were analyzed at the EAL along with bedding material and litter samples for TN content and M.C.

### 2.7. Live-Weight and Foot Health Examinations

**Figure 3:**
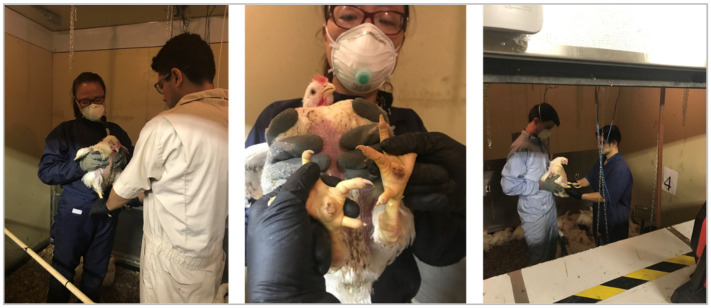
Visual inspections of broiler foot health for lesions and discoloration.

Birds were individually weighed and assessed for foot health, including evaluations of straightness, valgus deformities, valgus, FPD, and HB during early morning time at 28, 42, and 61 days of age. FPD assessments were conducted by inspecting the bottoms of birds’ footpads for lesions and discoloration. A scoring system of FPD ranging from 0–9 (0 = none; 9 = severe), adapted from Allain et al. [33], was used for this evaluation. HB was assessed by inspecting the hocks for lesions and discoloration, using a scoring system ranging from 0–3 (0 = none; 3 = severe). Figure 3 shows inspection of the leg health. Unpaired, unequal variance *t*-tests were performed to compare High and Low AV for each individual experiment as well as across the combined results of experiments 1–4.

## 3. Results

### 3.1. Chamber Environment Conditions

The AV treatments were applied based on the inlet T and the birds’ age. Birds were under heat-stressed conditions when the environment exceeded the “About optimum” level defined by Table 1. Table 3 shows that flocks 1, 2, 3, and 4 exceeded the optimal grow-out conditions by 72%, 54%, 57%, and 53% of the total experiment time, respectively. During flocks 1 and 3, the AV exceeded the life-threatening conditions more than flocks 2 and 4, which infers that flocks/experiments 1 and 3 were more stressful.

### 3.2. Litter Characteristics (TN, TAN, M.C., and pH)

As shown in Table 4, litter characteristics varied at the inlet and outlet of the chambers. The significant differences in M.C. at these locations were observed during the first and fourth experiments. Under both treatments, the outlet of the chamber observed significantly higher M.C. than that of the inlet. Likewise, the pH of the litter was significantly higher at the outlet than in the inlets. The entering air became warmer while leaving the chambers due to the added heat from the broilers. Therefore, the warmer air resulted in higher M.C. at outlets. Higher MC at outlets caused lower litter TN at the outlet in all cases except for the third experiment. These differences are likely caused by the broilers’ tendency to stay at the chamber inlets where AV was higher as air was entering the core chambers.

**Figure 4:**
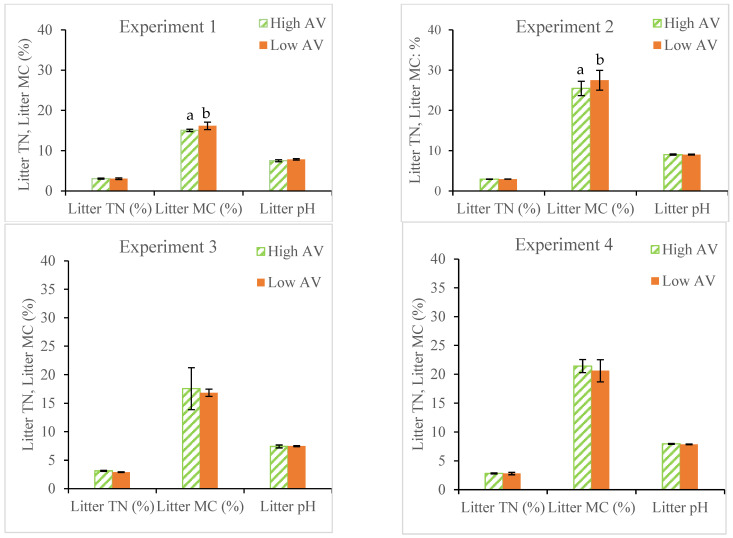
Comparisons of the mean AWAs (TN, M.C., pH) between the High and Low AV treatments within experiments. (There are significant differences in each litter characteristics between AV treatments displayed with different letters). (AV = air velocity, TN = total nitrogen, MC = moisture content, AWA = area-weighted average).

**Figure 5:**
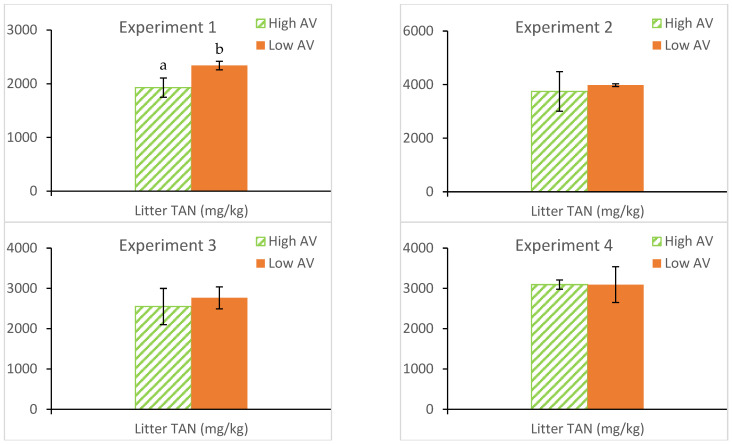
Comparisons of the mean AWAs (TAN) between High and Low AV treatments. (There are significant differences in litter TAN characteristics between AV treatments dis-played with different letters). (AV = air velocity, TAN = total ammoniacal nitrogen, AWA = area-weighted average).

Figure 4 and Figure 5 illustrate the comparisons of litter characteristics (i.e., TN, TAN, M.C., and pH) between High and Low AV treatments.

The AWAs of TN contents were not significantly different between AV treatments for experiments 1, 2, and 4. For experiment 3, the AWA (TN) was 7.90% higher (*p* < 0.05) in chambers receiving High AV than low AV (3.14 ± 0.1 vs. 2.91 ± 0.06%).

For AWA (TAN), a significant difference was only observed for experiment 1; the TAN was 19.22% lower (*p* < 0.05) in chambers receiving higher AV than Low AV (1928 ± 179 vs. 2338 ± 79 mg/kg). For AWA (M.C.), a significant difference was only observed in experiment 2; the M.C. was 9.47% lower (*p* < 0.05) in chambers receiving High AV than Low AV (25.46 ± 1.77 vs. 27.50 ± 2.47). The results in M.C. seem against our physical observations, in which much drier bedding under High AV treatment was observed as compared to those in Low AV treatments for all four experiments. Uncertainties may come from two sources: first, litter samples were stored in freezers for weeks at the EAL until they were analyzed for M.C. Duration and storage method may compromise M.C. results; second, due to the spatial variations, there might be a significant amount of uncertainty associated with AWA methods for calculating chamber-wide litter characteristics.

There was no significant difference in pH values between AV treatments for all four experiments.

### 3.3. NH_3_ Emissions

As shown in Table 5, in all the four experiments, NH_3_ emissions were lower under High AV than those under Low AV. Experiment 2 had the highest NH_3_ emission, whereas experiment 1 had the lowest emissions. The higher surface AV led to litter drying effects, thus reducing NH_3_ emissions in chambers under High AV. In addition, longer duration of higher AV seems to have resulted in less TAN available for volatilization and thus, less NH_3_ emissions during experiment 1. The negative NH_3_ emissions during experiment 3 at High AV in Table 5 may be attributed to uncertainties or variations in the nitrogen mass balance calculation. Specifically, the positioning of birds and their tendency to gather in certain areas, such as near inlets where AV was higher, could have influenced the spatial distribution of nitrogen in the litter. This uneven distribution could lead to uncertainty in calculating total nitrogen content in the litter leaving the chamber (N_litter_ in Equation (2)) with the AWA method. Moreover, High AV may have significantly dried the litter, reducing moisture content to levels that limit NH_3_ volatilization. This effect could result in lower emissions than expected or even negative values when uncertainties in measurements are factored in.

### 3.4. Heavy Broilers’ Performance Parameter

#### 3.4.1. Individual Body Weight and Foot Health

**Figure 6:**
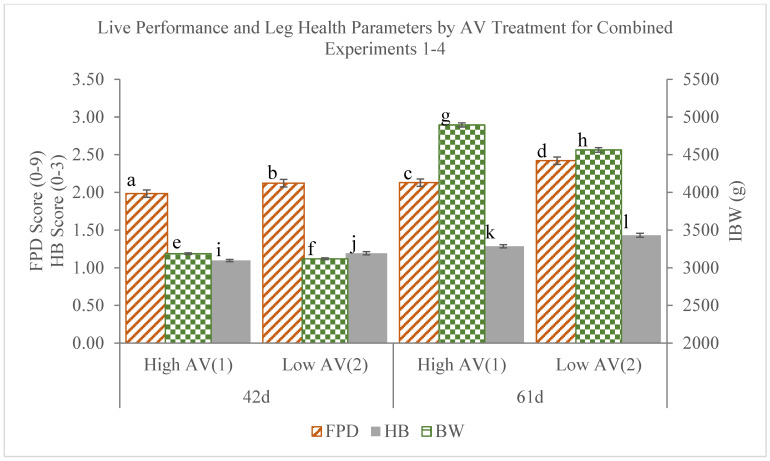
Comparisons of mean IBW, FPD, and HB between AV treatments on day 42 and day 61 for combined expts. 1–4 (Different subscripts for a given parameter between AV and day are considered significantly different) (AV = air velocity, IBW = individual body weight, FPD = footpad dermatitis, HB = hock burns).

Figure 6 summarizes data of live broiler individual body weight (IBW) as well as HB and FPD scores, averaged (avg. ± SE) by AV treatment and experiment at broiler ages of 42 days and 62 days. At both 42 and 61 days of age, the averaged FPD and HB scores were lower among those under High AV treatment than Low AV treatment.

The IBWs were lower among chambers receiving Low AV treatment than High AV treatment on both day 42 and day 61. Chambers receiving High AV treatment on day 42 generated 2.2% more IBW (*p*-value < 0.001) than chambers receiving Low AV treatment. Additionally, High AV treatment led to 6.5% and 8.1% improvements to FPD (*p*-value < 0.05) and HB (*p*-value < 0.001) scores. Chambers receiving High AV treatment on day 61 generated 7.3% more IBW (*p*-value < 0.001) than chambers receiving Low AV treatment. Additionally, AV1 treatment led to 12.1% and 10.2% improvements to FPD (*p*-value < 0.001) and HB (*p*-value < 0.001) scores. The differences among AV treatments between days were also compared and considered.

#### 3.4.2. Mortalities

**Figure 7:**
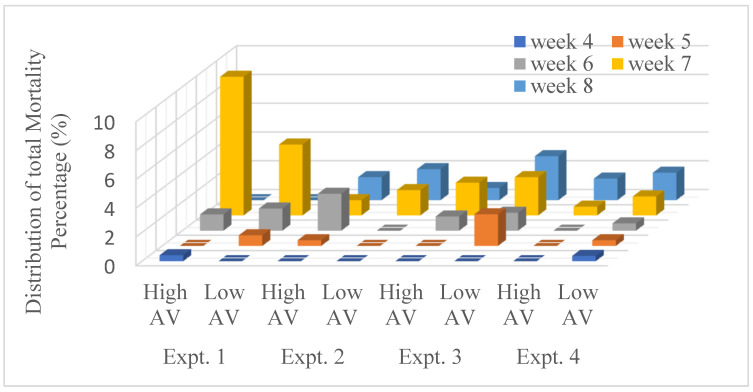
Chamber mortalities (%) by AV treatment and week for experiments 1–4.

The weekly average mortality percentage for Low and High AV treatments of all four experiments are shown in Figure 7. As expected, the mortality rates in experiment 1 were higher compared to the later flocks, as the first flock was more stressful than the other three. Due to the nature of T increase towards the end of summer, a higher mortality rate is seen in weeks 6 to 8 than in previous weeks for all four experiments. However, except for experiment 1, all other three experiments showed that higher AV treatment led to lower mortality rates.

## 4. Discussion

The results of the experiment provide valuable insights into the effects of AV treatment on heavy broiler performance and NH_3_ emissions under heat stress conditions. Notably, the measured litter characteristics showed significant differences between chamber inlets and outlets, suggesting that broilers tended to stay at the chamber inlets, where AV was highest.

The fact that early summertime in Raleigh, NC, tended to be hotter and drier than late summertime can not only be distinguished by viewing chamber T and RH but also by the amount of time maximum AV was implemented during grow-out. The AV reached its maximum intensity, i.e., during life-threatening and warning thermal conditions (Table 3) inside the chambers. Experiment 1 had more time reaching higher AV magnitude. Because of this, as expected, litter M.C. observed during experiment 1 was significantly lower than that of experiments 2 and 4, which may be a direct result of the ‘litter drying effect’. However, Mou et al. [34] found continuous air circulation of 0.76 m/s can dry litter effectively in younger birds (7–28 day old birds) [34]. Therefore, our experimental results extended the effectiveness of ventilation strategies across different life stages, more specifically at later ages.

The lowest (14.3–15.05%) and highest (27.99–29.04%) M.C. observed from the four experiments were from experiment 1 under High AV treatment and experiment 2 under Low AV treatment, respectively. As expected, the lowest M.C. resulted from higher AV treatment being administered for a greater amount of time. Similarly, it is no surprise that the highest M.C. was observed under the lower AV treatment, which experienced a lower duration of maximum AV treatment. This experiment conducted on heavy broilers were consistent with experiments for smaller birds, as Weaver et al. [35], who also observed an increased internal air movement significantly reduced the litter moisture and litter caking from a chamber study conducted on birds on their age from 14 to 42 d. At lower litter M.C., N compounds tend to remain adhered to the litter surface instead of volatilizing into the airstream above. Evidence of this can be seen in Table 5, with observations of TANs being greater in experiments 1 and 3 as opposed to experiments 2 and 4. Furthermore, litter TAN contents of experiments 1 and 3 were also notably lower than that of experiments 2 and 4. On one hand, this may be a result of fewer nitrogenous compounds converting to TAN. However, on the other hand, the greater amount of TAN in experiments 2 and 4 may be a result of administering less maximum AV treatment (e.g., less airflow at the litter surface resulting in less volatilization). The lower litter M.C. and NH_3_ emissions coupled with higher litter TN are evidence that the effect known as the “litter drying effect” is beneficial to heavy broiler production.

As the broilers grew heavier each week, they became less tolerant to heat; as a result, a higher mortality rate was observed towards the end of each experiment. Except for experiment 1, High AV resulted in lower mortality for all. This happened due to the experimental setup at the beginning of the experiment. During experiment 1, the AV treatment was not yet to be automated in response to the current environmental condition throughout the night. Hence the inability to increase the AV when the birds were experiencing heat stress resulted in higher mortality then. In later experiments, the AV treatments were fixed to be automated. The lower average IBW under Low AV infers the ability of High AV treatment to sufficiently reduce heat stress during the production cycle. Dozier et al. [35] examined the higher growth rate of broilers from 43 to 49 days of age due to an increased air velocity (AV) of 3 m/s, which improved (*p* ≤ 0.02) and provided evidence that increased AV is beneficial for broilers weighing 2.5 kg or greater when exposed to moderate temperatures. Further, at any age, FPD and HB scores can be decreased with High AV. Additionally, the mortality rates, particularly in the stressful conditions of experiment 1, were mitigated by higher AV treatment in subsequent experiments, emphasizing the role of AV in reducing mortality rates under heat stress conditions.

Furthermore, the study demonstrated that NH_3_ emissions were consistently lower under High AV compared to Low AV, with experiment 2 showing the highest NH_3_ emission and experiment 1 exhibiting the lowest. This reduction in NH_3_ emissions under High AV was attributed to the litter drying effects, indicating the potential environmental benefits of optimizing AV treatment.

## 5. Conclusions

Through four live broiler experiments under summer conditions, important information was obtained about heat stress impact and its mitigation on heavy broilers. High AV was found to be an effective medium to minimize heat stress impact by improving performance and welfare parameters of birds such as IBW, FPD, HB, or mortality. Higher AV treatment led to lower FPD and HB scores. Litter was drier with lower TAN in chambers receiving higher AV treatments as compared to those under lower AV treatments than in chambers receiving lower AV treatment. Litter pH became elevated when broilers were subjected to lower AV treatment for longer durations of time (as observed in experiment 2), resulting in higher litter M.C. and ultimately more NH_3_ emission from the litter surface. Broiler performance parameter IBW in chambers receiving higher AV treatment was significantly improved on day 42 and day 61 when compared to chambers receiving lower AV treatment on the same days. The findings underscore the importance of carefully tailored AV treatments in alleviating heat stress, improving litter conditions, reducing NH_3_ emissions, and enhancing overall broiler performance and welfare. This study contributes valuable information for the optimization of environmental conditions in broiler production systems, with implications for both animal well-being and environmental sustainability.

## Figures and Tables

**Table 1:** Live broiler heat stress experiments in the summers of 2017 and 2018.

Experiment	Bird-in Date	Bird-in Age	Bird-Out Date	Bird-Out Age
1	23 June 2017	23	1 August 2017	62
2	9 August 2017	28	12 September 2017	62
3	6 June 2018	28	10 July 2018	62
4	20 July 2018	28	23 August 2018	62

**Table 2:** High and low AV treatments for broiler experiments.

Treatment	Age (Days)	Below Optimum T		About Optimum T		Above Optimum T (Moderate)		Above Optimum T (Severe)		Above Optimum T (Life-Threatening)		Above Optimum T (Warning)	
		T °C	AV (m/s)	T °C	AV (m/s)	T °C	AV (m/s)	T °C	AV (m/s)	T °C	AV (m/s)	T °C	AV (m/s)
High	28–34 *	<26.0	0.9	26.0–27.8	1.23	27.8–28.9	1.33	28.9–32.2	1.48	32.2–33.9	1.64	>33.9	1.75
Low			0.9		1.23		1.33		1.48		1.64		1.75
High	35–40	<21.7	0.9	21.7–26.0	1.23	26.0–30.0	2.02	30.0–33.0	2.77	33.3–37.8	3.45	>37.8	3.95
Low			0.9		1.23		1.48		2.02		2.77		3.45
High	41–42	<21.1	1.48	21.1–26.0	1.48	26.0–30.0	2.02	30.0–33.0	2.77	33.3–37.8	3.45	>37.8	3.95
Low			1.48		1.48		1.48		2.02		2.77		3.45
High	43–52	<20.6	1.48	20.6–26.0	1.75	26.0–30.0	2.02	30.0–33.0	2.77	33.3–37.2	3.95	>37.8	4.33
Low			1.48		1.75		1.75		2.43		3.02		3.65
High	53–54	<19.4	1.48	19.4–25.0	1.75	25.0–29.5	2.43	29.4–32.7	3.02	32.7–36.1	3.95	>36.1	4.33
Low			1.48		1.48		1.75		2.43		3.02		3.65
High	55–56	<19.4	1.48	19.4–25.0	1.75	25.0–29.5	2.43	29.4–32.7	3.02	32.7–35.6	3.95	>35.6	4.33
Low			1.48		1.48		1.75		2.43		3.02		3.65
High	57–58	<18.9	1.48	18.9–25.0	1.75	25.0–29.5	2.43	29.9–32.2	3.02	32.2–35.6	3.95	>35.6	4.33
Low			1.48		1.48		1.75		2.43		3.02		3.65
High	59–60	<18.9	1.48	18.9–24.4	2.43	24.4–28.9	3.02	28.9–31.7	3.45	31.7–35.0	4.33	>35.0	4.43
Low			1.48		1.75		2.43		2.77		3.65		3.8
High	61	<18.3	1.48	18.3–23.9	2.43	23.9–28.9	3.02	28.9–31.7	3.45	31.1–33.9	4.33	>33.9	4.6
Low			1.48		1.75		2.43		2.77		3.65		3.95

* non-treatment period (in the first week) allowed broilers to acclimate to their new environment.

**Table 3:** Time distribution of AV treatment implemented from day 35 to day 61 in the four experiments.

Percentage of Occurrences from Total Observation							
Experiment	Below Optimum	About Optimum	Moderate	Severe	Life-Threatening	Warning	Exceeded Optimum Condition
1	0.08	28.03	36.77	19.88	14.77	0.46	71.89
2	1.18	45.34	35.45	13.03	5.00	0.00	53.48
3	1.70	41.30	35.47	13.48	7.93	0.11	57.00
4	0.00	46.63	40.02	13.34	0.00	0.00	53.37

**Table 4:** Comparison of measured litter characteristics at the core chamber inlets and outlets for High and Low AV during experiments 1–4.

Expt.	Treatment	Location	TN	M.C.	pH	TAN
			%	%		mg/kg WW
1	High	Outlet	2.54 ± 0.43 ^a^	17.24 ± 1.88 ^b^	8.24 ± 0.54 ^b^	2020 ± 560
		Inlet	3.41 ± 0.38 ^b^	11.87 ± 1.39 ^a^	6.75 ± 0.15 ^a^	1906 ± 138
	Low	Outlet	2.72 ± 0.36 ^a^	17.12 ± 2.48 ^b^	8.15 ± 0.32 ^b^	2339 ± 544
		Inlet	3.28 ± 0.13 ^b^	13.24 ± 0.88 ^a^	6.87 ± 0.17 ^a^	2101 ± 319
2	High	Outlet	2.42 ± 0.47 ^a^	25.93 ± 3.62	9.06 ± 0.35	3382 ± 856
		Inlet	3.41 ± 0.16 ^b^	22.77 ± 3.22	9.12 ± 0.11	3724 ± 1163
	Low	Outlet	2.31 ± 0.25 ^a^	23.84 ± 4.23	9.23 ± 0.15 ^b^	3529 ± 691 ^a^
		Inlet	3.01 ± 0.50 ^b^	26.66 ± 4.46	8.82 ± 0.41 ^a^	4456 ± 684 ^b^
3	High	Outlet	2.98 ± 0.77	17.63 ± 6.10	7.76 ± 0.43 ^b^	2486 ± 605
		Inlet	3.65 ± 0.85	16.02 ± 4.50	6.76 ± 0.17 ^a^	2270 ± 303
	Low	Outlet	2.40 ± 0.61 ^a^	20.91 ± 10.98	7.85 ± 0.27 ^b^	2957 ± 1056
		Inlet	3.12 ± 0.24 ^b^	13.88 ± 2.40	6.82 ± 0.09 ^a^	2432 ± 355
4	High	Outlet	2.42 ± 0.96 ^a^	22.98 ± 3.30 ^b^	8.24 ± 0.19 ^b^	2759 ± 589
		Inlet	3.26 ± 0.22 ^b^	17.39 ± 2.36 ^a^	7.30 ± 0.14 ^a^	2636 ± 263
	Low	Outlet	2.08 ± 0.64 ^a^	21.32 ± 3.59 ^b^	8.15 ± 0.24 ^b^	2925 ± 530
		Inlet	3.33 ± 0.23 ^b^	17.49 ± 2.82 ^a^	7.42 ± 0.11 ^a^	2870 ± 584

^a,b^ Means within treatments and columns with no common superscripts are different at (Pr |t| ≤ 0.01).

**Table 5:** Chamber environmental condition, frequency of maximum AV, and litter AWA (TN) based on NH_3_ emissions between AV treatments ^1,2,3,4,5^.

Expt.	Treatment	Median T	Median RH	AV_MAX_	M.C.	TAN	NH_3_
		°C	%	%	%	mg/kg	g/d
1	High	27.55	71.53	15.23	15.05 ± 0.30	1928 ± 179 ^a^	20.19 ± 13.35
	Low	27.55	71.53		16.18 ± 0.93	2338 ± 79 ^b^	30.58 ± 10.74
2	High	25.07	73.30	5.00	25.46 ± 1.77	3747 ± 738	40.48 ± 17.06
	Low	25.07	73.30		27.50 ± 2.47	3981 ± 52	59.03 ± 8.29
3	High	27.43	72.05	8.04	17.56 ± 3.67	2550 ± 448	−12.93 ± 4.39 ^a*^
	Low	27.43	72.05		16.85 ± 0.63	2765 ± 271	35.29 ± 1.56 ^b*^
4	High	26.13	82.91	0.00	21.42 ± 1.14	3092 ± 115	5.18 ± 2.48 ^a*^
	Low	26.13	82.91		20.61 ± 1.91	3092 ± 444	32.28 ± 3.23 ^b*^

^1^ T and RH: T and RH at the inlet location of the core chambers; ^2^ M.C., TAN, and NH_3_: mean ± SD; ^3^ Student’s *t*-tests were run between AV treatments for individual experiments; ^4^ a,b = (*p* < 0.05); a*,b* = (*p* < 0.01); ^5^ Previously reported M.C. and TAN columns serve as a discussion tool for NH_3_ emissions.

## Data Availability

The data presented in this study are available upon request from the corresponding author.
